# Ritonavir-Boosted Darunavir Is Rarely Associated with Nephrolithiasis Compared with Ritonavir-Boosted Atazanavir in HIV-Infected Patients

**DOI:** 10.1371/journal.pone.0077268

**Published:** 2013-10-10

**Authors:** Takeshi Nishijima, Yohei Hamada, Koji Watanabe, Hirokazu Komatsu, Ei Kinai, Kunihisa Tsukada, Katsuji Teruya, Hiroyuki Gatanaga, Yoshimi Kikuchi, Shinichi Oka

**Affiliations:** 1 AIDS Clinical Center, National Center for Global Health and Medicine, Tokyo, Japan; 2 Department of Community Care, Saku Central Hospital, Nagano, Japan; 3 Center for AIDS Research, Kumamoto University, Kumamoto, Japan; McGill University AIDS Centre, Canada

## Abstract

**Background:**

Although ritonavir-boosted atazanavir (ATV/r) is known to be associated with nephrolithiasis, little is known about the incidence of nephrolithiasis in patients treated with ritonavir-boosted Darunavir (DRV/r), the other preferred protease inhibitor.

**Methods:**

In a single-center cohort, the incidence of nephrolithiasis was compared between HIV-infected patients who commenced DRV/r-containing antiretroviral therapy and those on ATV/r. The effects of ATV/r use over DRV/r were estimated by univariate and multivariate Cox hazards models.

**Results:**

Renal stones were diagnosed in only one patient (0.86 per 1000 person-years) of the DRV/r group (n=540) and 37 (20.2 per 1000 person-years) of the ATV/r group (n=517). The median [interquartile (IQR)] observation period in the DRV/r group was 27.1 months (IQR 18.1-38.4 months), and 40.6 months (IQR 17.5-42.7) for the ATV/r group. The total observation period was 1,163.6 person-years and 1,829.6 person-years for the DRV/r group and for the ATV/r group, respectively. In the 37 patients on ATV/r who developed nephrolithiasis, the median time from commencement of ATV/r to diagnosis was 28.1 months (IQR 18.4–42.7), whereas nephrolithiasis in the single patient of the DRV/r group occurred 11.2 month after the introduction of DRV/r. ATV/r use over DRV/r was significantly associated with nephrolithiasis by uni- and multivariate analyses (HR=26.01; 95% CI, 3.541–191.0; p=0.001) (adjusted HR=21.47; 95% CI, 2.879–160.2; p=0.003).

**Conclusion:**

The incidence of nephrolithiasis was substantially lower in patients on DRV/r than those on ATV/r. The results suggest that DRV/r should be selected for treatment of HIV-infected patients at risk of chronic kidney disease.

## Introduction

Ritonavir-boosted darunavir (DRV/r) and ritonavir-boosted atazanavir (ATV/r) are the only two protease inhibitors (PI) selected as the preferred choices in the American Department of Health and Human Services (DHHS) guidelines for the initial treatment of patients infected with human immunodeficiency virus-1 (HIV-1) (http://www.aidsinfo.nih.gov/ContentFiles/AdultandAdolescentGL.pdf). Both drugs are widely used in combination with other antiretroviral drugs, based on their high efficacy, tolerability, favorable lipid profile, and once-daily dosing [1-4]. However, nephrolithiasis has been reported in patients receiving ATV/r-containing antiretroviral therapy (ART) [5,6]. Several case reports documented high concentrations of ATV in renal stones, suggesting the involvement of ATV in nephrolithiasis [5-8]. We recently reported in a single center cohort study that the incidence of renal stones is approximately 10 times higher among patients on ATV/r-containing antiretroviral therapy (ART) than those on other PIs-containing ART [9].

Our study on the effects of ART on renal stone formation included only a small number of patients on DRV/r-containing ART [9,10], and no data are available at present on the incidence of nephrolithiasis in patients treated with DRV/r. Of note, de Lastours et al [11] recently reported higher ATV and DRV levels in urine samples than in plasma, whereas plasma and urinary levels of lopinavir, another commonly used PI, were comparable. They also reported the presence of PI-containing crystals in the urine of a small proportion of patients on ATV and on DRV, but not on lopinavir/ritonavir (LPV/r). The data presented by de Lastours et al suggest that DRV can crystallize in urine leading to nephrolithiasis.

The aim of the present study was to determine the incidence of DRV/r- and ATV/r-related nephrolithiasis. Such comparison is important for two reasons: 1) These two PIs are most frequently prescribed PIs in resource-rich settings, and 2) nephrolithiasis is a risk factor for chronic kidney diseases (CKD) and end-stage renal disease (ESRD), which are important comorbidities associated with AIDS and death [12-16].

## Methods

### Ethics statement

This study was approved by the Human Research Ethics Committee of the National Center for Global Health and Medicine, Tokyo. Each participant provided a written informed consent for the clinical and laboratory data to be used and published for research purposes. The study was conducted according to the principles expressed in the Declaration of Helsinki.

### Study Subjects

We performed a retrospective, single-center cohort study of HIV-1-infected patients using the medical records kept at the National Center for Global Health and Medicine, Tokyo, Japan. Our facility is one of the largest clinics for patients with HIV infection in Japan with more than 2,700 registered patients. The study population was HIV-infected patients, aged >17 years, who commenced treatment with DRV/r or ATV/r-containing ART between January 1, 2004 and June 30, 2012. Both treatment-naïve and treatment-experienced patients were included. The follow-up period started at the time of commencement of ART containing the abovementioned drugs for the first time during the study period, and patients were followed until June 30, 2013. Patients were excluded if they had; 1) commenced the abovementioned ART during the study period at other facilities, 2) been prescribed unboosted ATV, or 3) been under treatment for nephrolithiasis at the time of commencement of the abovementioned ART. ATV/r became available in Japan in January 2004, and DRV/r in December 2007.

The attending physician selected either ATV/r or DRV/r at baseline. The use of these drugs was based on the Japanese guidelines, which placed ATV/r and DRV/r as the preferred choice, at least for 5 years during the study period (http://www.haart-support.jp/pdf/guideline2013.pdf. in Japanese). The attending physician also selected the concurrent drugs including nucleoside reverse transcriptase inhibitors (NRTI), non-NRTI, integrase inhibitors, and CCR5 inhibitors. None of the patients received two PIs during the study period.

### Measurements

The main investigator reviewed the medical records of all study patients to identify those with renal stones. Then two other investigators reviewed the set of medical records of each patient with renal stones to determine whether the case fitted into the following pre-defined criteria for nephrolithiasis: cases with a clinical diagnosis by the attending physician based on new onset of acute flank pain plus one of the following: 1) new-onset hematuria confirmed by urine dipstick test, 2) documented presence of stones or radiological findings suggestive of renal stones, such as hydronephrosis or obstruction or dilatation of the ureter, by either abdominal ultrasonography or computed tomography, 3) stone passage confirmed by either the patient or attending physician [9]. Patients with acute flank pain due to etiologies other than nephrolithiasis were excluded. At the time of diagnosis of nephrolithiasis, the attending physician selected either discontinuation or modification of ART. In our clinic, it is customary for the patient to visit the clinic once a month before the initiation of ART and until the suppression of HIV-1 viral load, but the visit interval is extended up to every three months after viral load suppression. 

In this study, the primary exposure variable was ATV/r use over DRV/r. The potential risk factors for nephrolithiasis were determined according to previous studies and collected from the medical records, together with the basic demographics [7,8,17]. They included age, sex, body weight, body mass index (BMI)={bodyweight (kg) / [(height (m)]^2^}, baseline laboratory data [CD4 cell count, HIV viral load, estimated glomerular filtration rate (eGFR), serum uric acid], and presence or absence of other medical conditions [concurrent use of tenofovir (TDF), past history of nephrolithiasis, previous exposure to indinavir (IDV), co-infection with hepatitis B defined by positive hepatitis B surface antigen, and co-infection with hepatitis C defined by positive hepatitis C viral load]. eGFR was calculated using the equation of the 4-variable Modification of Diet in Renal Diseases (MDRD) study [18]. For patients on ATV/r-containing ART, the value of serum total bilirubin was collected in two ways: for stone cases, total bilirubin value on the day was collected, and for non-stone cases, the value of total bilirubin 2 years after initiation of ATV/r was collected. For patients who discontinued ATV/r within 2 years, the value closest to the day of discontinuation was used. At our clinic, weight was measured on every visit whereas other variables were measured in the first visit and at least once annually. We used the data on or closest to and preceding the day of starting ART by no more than 180 days, except for serum uric acid level, which were collected within 180 days from the day of starting ART. 

### Statistical analysis

Baseline characteristics were compared using the Student's *t*-test or χ^2^ test (Fisher exact test) for continuous or categorical variables, respectively. The time to the diagnosis of nephrolithiasis was calculated from the date of commencement of DRV/r- or ATV/r-containing ART to the date of diagnosis of nephrolithiasis. Censored cases represented those who discontinued ATV/r or DRV/r, dropped out, were referred to other facilities, or at the end of follow-up period. The time from the start of ART to the diagnosis of nephrolithiasis was analyzed by the Kaplan Meier method for patients who started DRV/r (DRV/r group) and ATV/r (ATV/r group), and the log-rank test was used to determine the statistical significance. The Cox proportional hazards regression analysis was used to estimate the impact of ATV/r use over DRV/r on the incidence of nephrolithiasis. The impact of each basic demographic parameter, baseline laboratory data, and other medical conditions listed above was also estimated with univariate Cox proportional hazards regression. To estimate the unbiased prognostic impact of ATV/r use over DRV/r for nephrolithiasis, we conducted three models using multivariate Cox proportional hazards regression analysis. Model 1 was the aforementioned univariate analysis for ATV/r use over DRV/r. Model 2 included age, sex, and weight plus model 1 in order to adjust for basic characteristics. In model 3, we added variables with P values <0.05 in univariate analysis after adjustment (these included tenofovir use, serum uric acid per 1 mg/dl, and past history of renal stones). Possible risk factors for ATV/r-related nephrolithiasis identified in previous studies were also added to model 3 (these included prior exposure to IDV) [7,8].

In addition, to examine the impact of serum total bilirubin on ATV/r-containing ART and the incidence of nephrolithiasis, the median serum total bilirubin values were compared between the renal stone and non-renal stone groups using the Mann-Whitney U test. 

Statistical significance was defined as two-sided *p* values <0.05. We used hazard ratios (HRs) and 95% confidence intervals (95%CIs) to estimate the impact of each variable on nephrolithiasis. All statistical analyses were performed with The Statistical Package for Social Sciences ver. 20.0 (SPSS, Chicago, IL).

## Results

**Figure 1:**
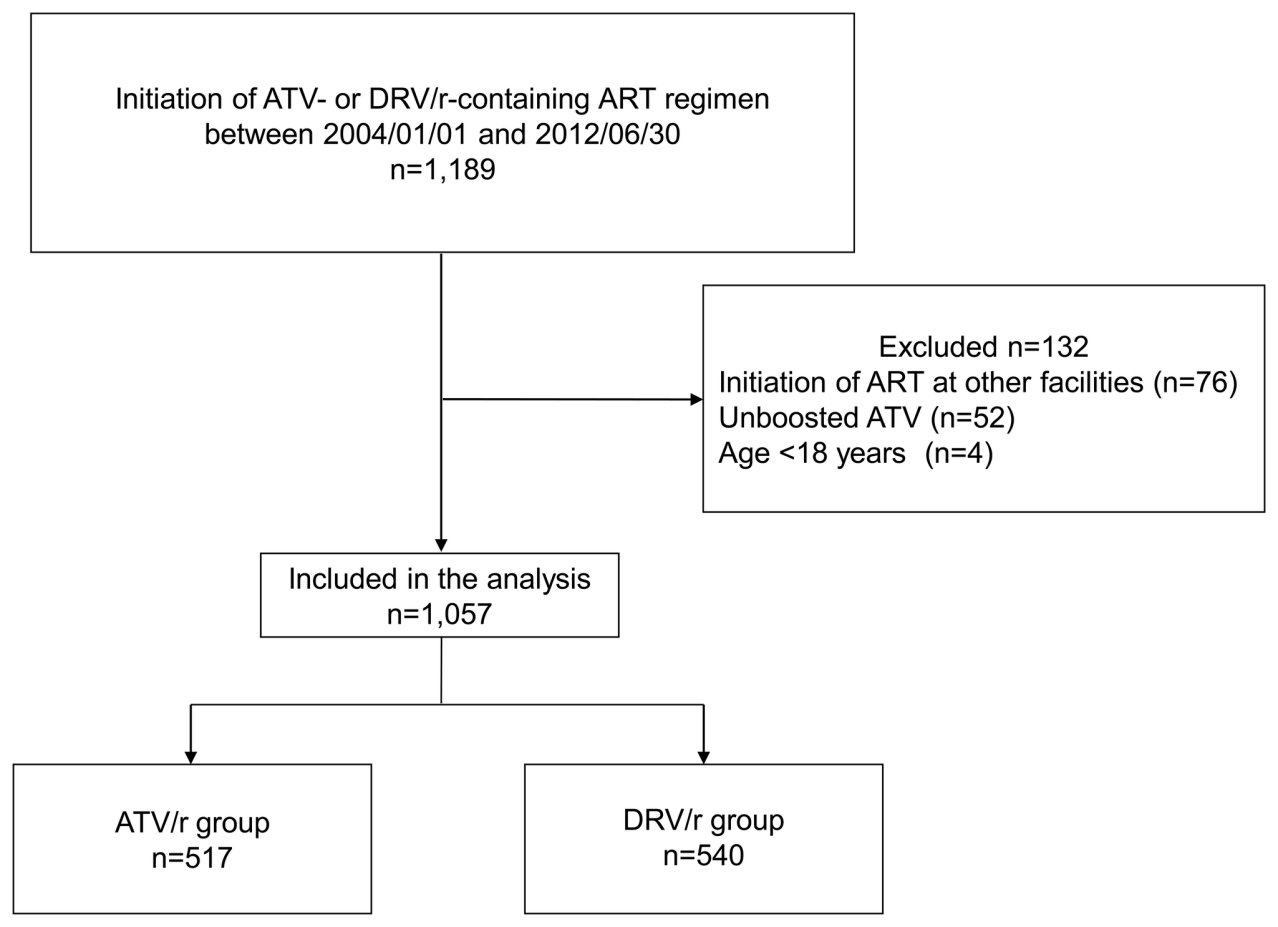
Flow diagram of patient selection. ART, antiretroviral therapy; ATV, atazanavir; DRV/r, ritonavir-boosted darunavir; ATV/r, ritonavir-boosted atazanavir.

A total of 1,189 patients commenced either DRV/r- or ATV-containing ART between January 1, 2004 and June 30, 2012. Of the 1,057 patients who were included in the analysis, 540 (51%) started DRV/r-containing ART while 517 (48.9%) started ATV/r-containing ART (Figure 1). Table 1 shows the baseline characteristics of the study population. The ATV/r group included significantly younger (p=0.019), more patients of East Asian origin (p=0.009) with higher BMI (p=0.014), higher CD4 count (p=0.038), higher baseline serum uric acid (p=0.007), and a larger proportion of patients with past history of urinary stones (p=0.017) and previous exposure to IDV (p=0.036). In contrast, patients of the DRV/r group were significantly more likely to use tenofovir (p <0.001) and with higher viral load (p=0.002) (Table 1).

**Table 1:** Baseline demographics and laboratory data of patients who received ritonavir-boosted darunavir- or ritonavir-boosted atazanavir-containing antiretroviral therapy.

	DRV/r (n=540)	ATV/r (n=517)	P^a^
Age, years*	39 (33-46)	36 (31-44)	0.019
Male sex	498 (92.2)	480 (92.8)	0.727
Race (East Asian origin)	494 (91.5)	494 (95.6)	0.009
Body weight, kg*	62.1 (55.8-70)	64.0 (57.6-72)	0.074
body mass index, kg/m^2^*	21.7 (19.8-24.1)	22.4 (20.4-24.6)	0.014
CD4 cell count, cells/μL*	251 (90-399)	260 (190-383)	0.038
HIV load, log_10_ copies/mL*	4.27 (1.70-5.17)	3.94 (1.70-4.66)	0.002
Treatment naïve	309 (57.2)	280 (54.2)	0.322
Tenofovir use	342 (63.3)	196 (37.9)	<0.001
eGFR, mL/min/1.73 m^2^*	116 (102-131)	115 (103-130)	0.842
Serum uric acid, mg/dL*	5.7 (4.7-6.5)	5.9 (5.1-6.7)	0.007
HBV or HCV coinfection	78 (14.4)	64 (12.4)	0.367
Past history of nephrolithiasis	22 (4.1)	39 (7.5)	0.017
Previous exposure to IDV	25 (4.6)	41 (7.9)	0.030

Data are number (%) of patients or * median (interquartile range).

DRV/r, ritonavir-boosted darunavir; ATV/r, ritonavir-boosted atazanavir; eGFR, estimated glomerular filtration rate; HBV, hepatitis B virus, HCV, hepatitis C virus, HIV, human immunodeficiency virus; IDV, indinavir.

a The χ^2^ test or Fisher exact test was used for categorical data, and the Student *t* test was used for continuous variables.

Thirty eight patients fulfilled the pre-defined criteria for nephrolithiasis. Nephrolithiasis was identified in 1 (0.2%) of the DRV/r group and 37 patients (7.1%) of the ATV/r group, with an estimated incidence of 0.86 and 20.2 per 1,000 person-years, respectively. The incidence of nephrolithiasis in the ATV/r group was approximately 20 times higher than that in the DRV/r group. 

**Figure 2:**
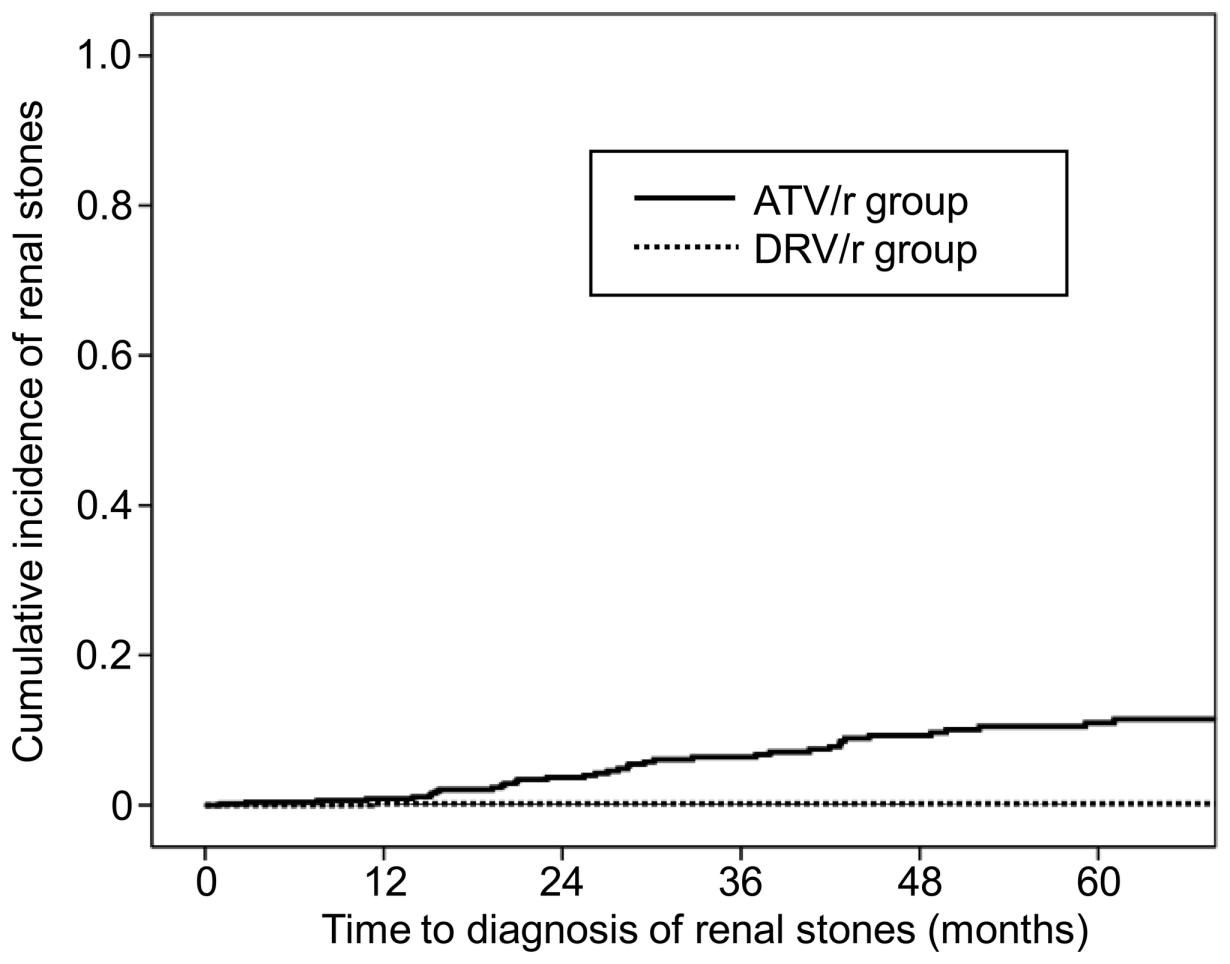
Kaplan-Meier curve showing time to the diagnosis of nephrolithiasis. ATV/r, ritonavir-boosted atazanavir; DRV/r, ritonavir-boosted darunavir.

Of the patients with nephrolithiasis, 9 and 12 were diagnosed by hematuria and stone passage, respectively, as defined above. Furthermore, 17 were diagnosed by radiological studies, of which renal calcification was identified in 5 patients. Figure 2 shows the time from initiation or switching of DRV/r or ATV/r to the diagnosis of nephrolithiasis by the Kaplan Meier method. Patients of the ATV/r group were significantly more likely to develop renal stones, compared to those of the DRV/r group (p<0.001, Log-rank test). 

The median time from the commencement of ART to the diagnosis of nephrolithiasis was 28.1 months [interquartile range (IQR), 18.4–42.7 months] for the ATV/r group and only one patient with nephrolithiasis in the DRV/r group was diagnosed 11.2 month after the introduction of DRV/r-containing ART. The total observation period was 1,163.6 patient-years [median, 27.1 months, IQR, 18.1–38.4 months] for the DRV/r group, and 1,829.6 patient-years [median, 40.6 months, IQR, 17.5–42.7 months] for the ATV/r group. Among the ATV/r group, the median total bilirubin value of the renal stone group was marginally higher than that of the non-renal stone group [2.7 (IQR 2-3.8) and 2.2 mg/dl (IQR 1.6-3.0), respectively, P=0.051].

Univariate analysis showed a significant relationship between ATV/r use and nephrolithiasis (HR=26.01; 95% CI, 3.541–191.0; p=0.001) (Table 2). Higher serum uric acid (HR=1.415; 95% CI, 1.173–1.705; p<0.001) and past history of nephrolithiasis (HR=2.658; 95% CI, 1.111–6.359; p=0.028) were also significantly associated with the nephrolithiasis. On the other hand, tenofovir use was negatively associated with nephrolithiasis (HR=0.435; 95% CI, 0.210–0.899; p=0.025) (Table 2). Multivariate analysis identified ATV/r use over DRV/r as an independent risk for nephrolithiasis after adjustment for age, male sex, and weight (adjusted HR=27.08 95% CI, 3.680–199.3; p=0.001) (Table 3, Model 2), and also after adjustment for other risk factors (adjusted HR= 21.47; 95% CI, 2.879–160.2; p=0.003) (Table 3, Model 3).

**Table 2:** Univariate analysis to estimate the risk of various factors for nephrolithiasis.

	Hazard ratio	95%CI	P value
ATV/r use over DRV/r	26.01	3.541-191.0	0.001
Age per 1 year	1.002	0.973-1.031	0.907
Male sex	1.665	0.401-6.919	0.483
Race (East Asian origin)	2.287	0.314-16.68	0.414
Weight per 1 kg increment	0.998	0.970-1.027	0.872
body mass index per 1 kg/m^2^ increment	0.996	0.905-1.095	0.927
CD4 count per 10 /μl increment	0.999	0.983-1.016	0.901
HIV viral load per log10/ml	1.063	0.859-1.316	0.575
Treatment naïve	1.020	0.538-1.936	0.950
Tenofovir use	0.435	0.210-0.899	0.025
Baseline eGFR per 10 ml/min/1.73 m^2^ decrement	1.103	0.980-1.242	0.105
Baseline serum uric acid per 1 mg/dl increment	1.415	1.173-1.705	<0.001
Hepatitis B or Hepatitis C	1.418	0.592-3.393	0.433
Past history of renal stone	2.658	1.111-6.359	0.028
Previous exposure to IDV	1.192	0.366-3.879	0.771

ATV/r, ritonavir-boosted atazanavir; DRV/r, ritonavir-boosted darunavir; HIV, human immunodeficiency virus; eGFR, estimated glomerular filtration rate; IDV, indinavir.

**Table 3:** Multivariate analysis to estimate the risk of ATV/r- over DRV/r-containing antiretroviral therapies for nephrolithiasis.

	Model 1 crude (n=1,057)		Model 2 adjusted (n=1,056)		Model 3 adjusted (n=1,021)	
	HR	95%CI	HR	95%CI	HR	95%CI
ATV/r use over DRV/r	27.05	3.687-198.5	27.08	3.680-199.3	21.47	2.879-160.2
Age per 1 year			1.009	0.980-1.039	1.006	0.976-1.037
Male sex			1.939	0.441-8.528	1.202	0.262-5.512
Weight per 1 kg increment			0.988	0.956-1.021	0.979	0.947-1.012
Tenofovir use					0.678	0.313-1.470
Baseline serum uric acid per 1mg/dl increment					1.418	1.150-1.750
Past history of renal stone					1.661	0.520-5.307
Past exposure to IDV					0.491	0.100-2.403

HR, Hazard ratio; CI, confidence interval; ATV/r, ritonavir-boosted atazanavir; DRV/r, ritonavir-boosted darunavir; HIV, IDV, indinavir.

The chemical composition of the renal stones of the single case on DRV/r was analyzed with high performance liquid chromatography with ultraviolet detection (HPLC-UV) method as described elsewhere [19,20], but the analysis did not identify DRV. Renal stones of patients on ATV/r were not analyzed.

## Discussion

To our knowledge, this is the first study that investigated the incidence of DRV/r-associated nephrolithiasis. Only a single case of nephrolithiasis was detected among 540 patients on DRV/r-containing ART with total observation period of 1,163.6 patient-years. The incidence of nephrolithiasis in the DRV/r group was only 0.86 per 1,000 person-years, comparable to that in the general population in Japan (1.14 per 1,000 person-years) [21], whereas that in the ATV/r group was 20.2 per 1,000 person-years, approximately 20 times higher. Univariate and multivariate analyses identified ATV/r use over DRV/r as an independent risk factor for nephrolithiasis with a high hazard ratio. Furthermore, in the single patient with nephrolithiasis on DRV/r, DRV was not detected as a component of renal stones. 

This study showed that the risk of nephrolithiasis is substantially lower among patients on DRV/r- than those on ATV/r-containing ART based on clinically feasible criteria. This finding is important considering DRV/r and ATV/r are the two PIs considered the preferred regimen for the treatment-naïve patients (http://www.aidsinfo.nih.gov/ContentFiles/AdultandAdolescentGL.pdf). Both PIs have similar characteristics; they are highly effective and tolerable with favorable lipid profile, and possess a high barrier to drug resistance [1-4]. One of the strengths of ATV/r is more abundant clinical evidence due to longer market availability than that of DRV/r. On the other hand, ATV/r often causes indirect hyperbilirubinemia, and requires acidic gastric environment for optimal absorption that requires some consideration on drug-drug interactions (http://www.aidsinfo.nih.gov/ContentFiles/AdultandAdolescentGL.pdf) (http://packageinserts.bms.com/pi/pi_reyataz.pdf). The substantially lower incidence of renal stones in patients on DRV/r than ATV/r adds another dimension to patient management in relation to the selection of a PI.

The development of renal stones, even a single episode, is a risk factor for CKD, doubling of serum creatinine level, and ESRD [12,13,16]. Many studies have also demonstrated that ATV/r use is a risk for renal dysfunction and CKD [22-25]. The high incidence of nephrolithiasis with ATV/r use identified in the present study may in part explain the risk of ATV/r for CKD. Thus, ATV/r should be introduced carefully in patients with concomitant predisposing factors for CKD. In this regard, there are no studies that show the association of DRV/r use with renal dysfunction or CKD, although this may in part be due to more recent introduction of DRV/r compared with ATV/r.

Why is nephrolithiasis less likely to occur with DRV/r compared to ATV/r? Although the mechanism of PI-induced nephrolithiasis is not fully understood, precipitation of pure PI is suggested as a possible etiology [8]. Up to 20% of IDV (an old PI well-known for its precipitation and renal stone formation) is excreted unchanged in the urine, a property that contributes to the high incidence of nephrolithiasis in patients treated with IDV [26] (http://www.merck.com/product/usa/pi_circulars/c/crixivan/crixivan_pi.pdf). Unchanged DRV and ATV are reported to be excreted in urine at similar proportions of 7.7% and 7% of the administered dose, respectively (http://packageinserts.bms.com/pi/pi_reyataz.pdf) (http://www.merck.com/product/usa/pi_circulars/c/crixivan/crixivan_pi.pdf). However, strong acidity (e.g., pH of 1.9) is required to achieve optimal dissolution of ATV, and its solubility in urine is known to decrease with increase in pH (http://packageinserts.bms.com/pi/pi_reyataz.pdf). Because urine is usually mildly acidic [9], the difference in the solubility of DRV and ATV in urine might explain the different incidence of nephrolithiasis in patients using these two PIs. Although de Lastours et al [11] described the presence of DRV crystals in the urine of 4 (7.8%) out of 51 patients on DRV/r and suggested that DRV/r use might be a risk for renal stones, the number of enrolled patients in their study was relatively small to allow firm conclusions. 

The present study has several limitations. First, due to the retrospective nature of the study, the baseline characteristics of the enrolled patients were not controlled. It is possible that more patients with potential risks for nephrolithiasis were included in the ATV/r group. In the ATV/r group, more patients were hyperuricemic, had history of renal stones, and previous exposure to IDV, which are known risk factors for nephrolithiasis. However, multivariate analysis clearly showed that ATV/r use is an independent risk factor with high hazard ratio even after adjustment for variables including the above three. Second, the median observation period was longer in the ATV/r group than in the DRV/r group (40.6 versus 27.1 months), suggesting that the risk of nephrolithiasis in the ATV/r group could be overestimated. Further studies are warranted to elucidate whether much longer use of DRV/r induces nephrolithiasis. However, it is noteworthy that in patients with nephrolithiasis, the median time from the commencement of ATV/r or DRV/r to the diagnosis of nephrolithiasis was 28.1 months (IQR: 18.4-42.7 months), which was similar to that of the DRV/r group [median 27.1 (IQR: 18.1-38.4)], backing up the result of the present study: the risk of nephrolithiasis is substantially lower among patients on DRV/r than those on ATV/r. Third, stone composition analysis was conducted in only one patient with renal stones (treated with DRV/r), therefore, it is possible that renal stones caused by other etiologies are included. 

In conclusion, the present study demonstrated that the risk of nephrolithiasis, an important risk factor of CKD, is approximately 20 times lower among patients on DRV/r- than those on ATV/r-containing ART, providing DRV/r one advantage over ATV/r in the selection of PI. ATV/r use was identified as a significant and independent risk factor for nephrolithiasis in a robust statistical model that included ATV/r use over DRV/r as a primary exposure. ATV/r should be prescribed with caution in patients with predisposing factors for nephrolithiasis and those with CKD.
